# Substitution of Proline Residues by 4-Fluoro-l-Proline Affects the Mechanism of the Proline-Rich Antimicrobial Peptide Api137 †

**DOI:** 10.3390/antibiotics14060566

**Published:** 2025-05-31

**Authors:** Maren Reepmeyer, Andor Krizsan, Alexandra Brakel, Lisa Kolano, Jakob Gasse, Benjamin W. Husselbee, Andrea J. Robinson, Ralf Hoffmann

**Affiliations:** 1 Institute of Bioanalytical Chemistry, Faculty of Chemistry and Mineralogy, Leipzig University, Deutscher Platz 5, 04103 Leipzig, Germanyandor.krizsan@uni-leipzig.de (A.K.);; 2 Center for Biotechnology and Biomedicine (BBZ), Leipzig University, Deutscher Platz 5, 04103 Leipzig, Germany; 3 School of Chemistry, Monash University, Wellington Road, Clayton, Melbourne, VIC 3800, Australia

**Keywords:** PrAMP, Api137, Fluoro-l-proline, mode of action

## Abstract

**Background**: The well-studied 18-residue-long proline-rich antimicrobial designer peptide Api137 utilizes at least two lethal intracellular mechanisms that target the bacterial 70S ribosome. First, Api137 stalls the ribosome by binding to the peptidyl-transferase center, trapping the release factor, and inhibiting protein expression. Second, Api137 disrupts the assembly of the large 50S subunit of the ribosome, resulting in partially assembled pre-50S dead-end particles that are unable to form the functional 70S ribosome. **Methods**: All six proline residues in Api137 were substituted with 4*S*- and 4*R*-fluoro-l-proline (Fpr), which promote the *cis*- and *trans*-conformer ratio of the preceding Xaa-Pro-bond, respectively. The effect on the antibacterial activity was studied using *Escherichia coli*. The underlying mechanisms were investigated by studying 70S ribosome binding, inhibition of *in vitro* translation, and ribosome profile analysis. **Results**: Interestingly, the analogs were equipotent to Api137, except for the 4*S*-Fpr11 and 4*S*-Fpr16 analogs, which were four times more or less active, respectively. The most active 4*S*-Fpr11 analog competed the least with Api137 for its ribosome binding site, suggesting a shifted binding site. Both Fpr14 and the 4*S*-Fpr16 analogs disturbed 50S subunit assembly less than Api137 or not at all. The strongest effect was observed with the 4*R*-Fpr16 analog resulting in the lowest 70S ribosome content and the highest pre-50S particle content. This peptide also showed the strongest competition with Api137 for its binding site. However, its antibacterial activity was similar to that of Api137, possibly due to its slower cellular uptake. **Conclusions**: Api137 inhibits protein translation and disrupts 50S assembly, which can be adjusted by substituting specific proline residues with fluoroproline. 4*R*-Fpr16 potently inhibits ribosome assembly and offers a novel, unexploited clinical mechanism for future antibiotic development.

## 1. Introduction

Across the last four decades, many peptides with antimicrobial activities have been reported; currently, 5099 antimicrobial peptides (AMPs) are listed in the Antimicrobial Peptide Database, of which 3306 are natural peptides (https://aps.unmc.edu/home, accessed on 18 March 2025) [1]. AMPs are produced in all kingdoms of life as part of host defense, protecting against pathogens and enhancing the immune response [2,3]. Over the past 30 years, research on AMPs has focused on the discovery of new derivatives, the determination of the mode of action, and optimization for clinical approaches such as sequence optimization to improve proteolytic stability and antimicrobial activity and to reduce cytotoxicity to human cells. Such pharmacologically optimized AMPs could provide an opportunity for the development of new antibiotic agents to fight resistant pathogens [1,4].

While most AMPs use a lytic mode of action, which typically results in low safety margins, proline-rich AMPs (PrAMPs) act via an intracellular mechanism [5,6,7] following a SbmA transporter-mediated bacterial uptake [8]. The primary target of PrAMPs is the bacterial ribosome to inhibit protein synthesis, although PrAMPs can interfere with protein folding by binding to the heat shock protein DnaK and the chaperone GroEL [9,10,11,12]. The bacterial ribosome, composed of three strands of ribosomal RNAs (rRNAs) and 54 ribosomal proteins, contains multiple functional centers for translating messenger RNA (mRNA) sequences into the encoded polypeptide chain [13,14,15,16]. Protein expression and ribosome assembly are highly complex and dynamic processes involving additional enzymes and ribosomal factors [16,17,18,19] that can theoretically be inhibited at different stages, as confirmed for PrAMPs from different species [7,20]. A frequently studied PrAMP lead structure is the designer peptide Api137 (gu-ONNRPVYIPRPRPPHPRL-OH) [21], which is derived from apidaecin 1b of the honeybee *Apis mellifera* [22]. It provides improved protease stability and antibacterial activity against some Gram-negative human pathogens, such as *Escherichia coli* [9,21].

The first mechanism described for Api137 was binding to the polypeptide exit tunnel (PET) of the 50S subunit (50S) near the peptidyl transferase center (PTC) of the 70S ribosome (“PTC binding site”) [20,23,24,25]. Api137 enters the PET and interacts with the ribosomal (23S) RNA, the ribosomal proteins uL4 and uL22, the deacylated peptidyl-site (P-site) tRNA, and the release factors 1 or 2 (RF1/RF2). Trapping the release factors, which are present in much smaller numbers than ribosomes, in a complex with Api137 depletes the pool of free release factors in the cytoplasm and stalls other ribosomes with their nascent protein chains in the termination step [20]. Recently, a second binding site has been identified within the 50S subunit in proximity to the exit of the PET (“PET exit binding site”), where the *N*-terminal peptide region of Api137 is positioned closer to the exit pore near the ribosomal proteins uL29 and uL23, while the *C*-terminal end reaches further into the PET close to uL22 [26,27]. A second independent mechanism, reported together with the first mechanism by Krizsan et al. in 2015 [7,28], relates to the effect of Api137 on disrupting ribosome assembly, as suggested by the presence of partially assembled large subunits at higher levels. In a recent study, it was verified that incubation of *E. coli* with Api137 during the exponential growth phase induced elevated levels of several 50S precursors compared to untreated *E. coli* [27]. Structural data showed that Api137 binds near uL22 [29], supporting the hypothesis that Api137 disrupts the early assembly process by preventing the binding of uL22 in the early assembly phase, leading to 50S precursors and dead-end particles.

Previous substitution studies on apidaecin and its analogs and recent cryo-EM data have highlighted the importance of the penultimate amino acid, Arg17 [20,21,30]. All substitutions tested so far at this position decreased or abolished the activity, whereas substitutions at other positions of the pharmacophore unit PHPRL are better tolerated. Even small structural changes in Api137, such as *C*-terminal amidation or single amino acid substitutions, typically affect the antimicrobial activity and binding behavior [20,21,30]. This was even true when one or several of the six proline residues in Api137 were substituted by *trans*-4-hydroxy-l-proline [21,30], whereas substitution of proline residues 3, 5, 10, and 14 on non-glycosylated drosocin with *trans*-4-hydroxy-l-proline improved the activity and the serum stability partially [31].

**Figure 1:**
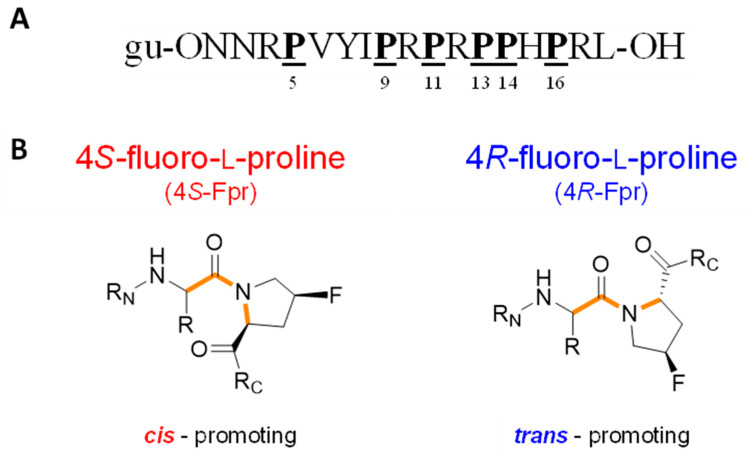
Structures of Api137 (**A**), 4*S*-fluoro-l-proline and 4*R*-fluoro-l-proline (**B**). Proline positions substituted with a fluoroproline in this study are underlined. Fluoroprolines are shown in the Natta projection. The color codes for 4*S*- (red) and 4*R*-fluoro-l-proline (blue), which favor the *cis*- and *trans*-equilibrium of prolyl-peptide bonds (orange), respectively, are used throughout this publication to show the data of the corresponding Api137 analogs.

As an alternative to *cis*- and *trans*-4-hydroxy-l-proline, we substituted the proline residues in the ribosomal binding region of Api137 with 4*S*-fluoro-l-proline (4*S*-Fpr) and 4*R*-fluoro-l-proline (4*R*-Fpr), which stabilize the *cis*- or *trans*-conformer of the preceding Xaa-Pro bond (Figure 1), respectively, accelerate the *cis*/*trans* prolyl peptide bond isomerization [32,33], and could influence binding characteristics due to the strong inductive effect and weak hydrogen-bond acceptance [33,34,35].

Previous studies have focused on the MIC or effects on protein translation and consequent effects when evaluating Api137 substitutes, overlooking the second mechanism of Api137, which has recently been described in detail [27,36,37]. This study investigates the preference of a *cis* or *trans* preferred peptide bond orientation preceding Fpr, as even small changes in the pharmacophore domain of Api137 can affect both target binding and bacterial uptake. A comparison of the inhibition constants (K_i_) and activities obtained for 4*S*-Fpr and 4*R*-Fpr should show whether a promoted conformation improves or worsens the desired antibacterial activity, allowing further structural optimization.

## 2. Results

All peptides (Table 1) were synthesized with typical yields ranging from 2.9 mg (10%) to 8.9 mg (31%) from a 12.5 µmol batch and a purity ranging from 91% to 98%. The Fmoc-fluoroproline derivatives appeared to be as reactive as Fmoc-Pro-OH.

### 2.1. Antimicrobial Activity Against Escherichia coli

**Figure 2:**
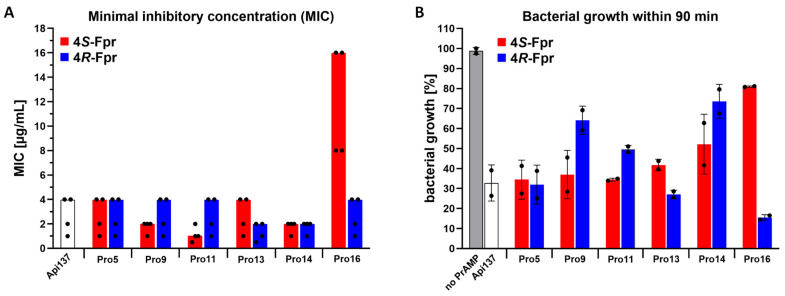
Effect of fluoroproline substitutions on the antibacterial activity of Api137 against *E. coli* RN31 tested after ~16 h (**A**) and 90 min (**B**). Color code of bars: white for Api137, red for 4*S*-Fpr, blue for 4*R*-Fpr, and gray for untreated control. (**A**), MICs were determined against an *E. coli* RN31 strain expressing a hybrid protein consisting of bS20 (30S) with mCherry and bL19 (50S) with EGFP in 33% TSB (bars) in two independent experiments with two replicates each (*n* = 4, dots). (**B**), Growth inhibition was determined for *E. coli* RN31 in 33% TSB at a peptide concentration of 8 µg/mL after incubation for 90 min. Values were normalized to the OD_600_ observed for an untreated *E. coli* RN31 culture. Shown are the mean values (bars) obtained from two biological replicates (dots) and the error bars (*n* = 2).

The minimum inhibitory concentration (MIC) of Api137 observed for *E. coli* RN31 after an incubation period of approximately 16 h was identical to previous reports (4 µg/mL) [27]. Substitution of most proline residues did not affect peptide activity with MICs of 2–4 µg/mL, except for 4*S*-Fpr at positions 16 (16 µg/mL) and 11 (1 µg/mL) (Figure 2A).

The immediate effects of the peptides on bacterial growth during the 90 min exponential growth phase of *E. coli* RN31 were tested at a peptide concentration of 8 µg/mL (Figure 2B), as in previous reports [27]. Compared to an untreated control, Api137 reduced bacterial growth by 70%, as did the substitution of Pro5. Substitution of Pro9 or Pro11 by 4*R*-Fpr only inhibited growth by 34% and 49%, respectively, whereas 4*S*-Fpr reduced growth by 62% and 65%, respectively, similar to Api137. When Pro13 was substituted, 4*S*-Fpr reduced growth by 56% and 4*R*-Fpr by 70%. Stronger effects were observed for the *C*-terminal positions. Both fluoroprolines at position 14 significantly decreased activity. Interestingly, substitution of Pro16 with 4*S*-Fpr had almost no effect on bacterial growth during the first 90 min (16% reduction), whereas substitution with 4*R*-Fpr very strongly inhibited growth by 84% compared to the control, corresponding to a slight increase in cell count (OD_600_ = 0.333 ± 0.002) of ~140% relative to the initial cell count (OD_600_ = 0.226 ± 0.006) at 0 min.

### 2.2. Ribosome Binding and Inhibition of In Vitro Translation

**Figure 3:**
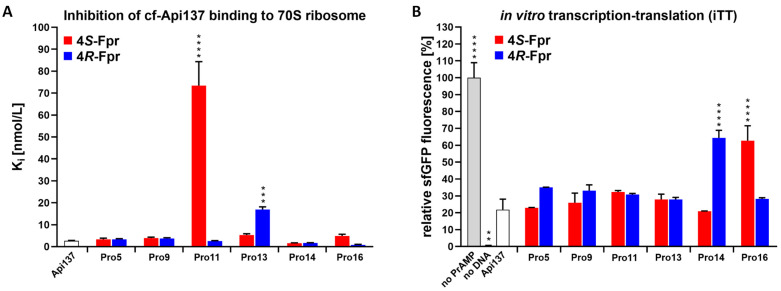
Effects of Api137 (white bar), 4*S*-Fpr-substituted Api137 (red), and 4*R*-Fpr-substituted Api137 (blue) on the interaction with 70S ribosome and translation. (**A**) K_i_-values obtained from competitive binding studies of cf-Api137 to crude ribosome in competition with unlabeled peptides. Error bars represent the deviation from the mean of two independent experiments with two replicates each (*n* = 4). Significance levels versus Api137 are indicated as *** (*p* value < 0.001) and **** (*p* value < 0.0001) by one-way ANOVA with Tukey’s multiple comparison test (GraphPad Prism, 10.4.1). (**B**) *In vitro* expression of sfGFP in the presence of RF1. Values were normalized to fluorescence recorded in the absence of PrAMPs (gray). SfGFP production without adding the sfGFP DNA template (no DNA, black). Error bars represent the deviation from the mean of three independent experiments (*n* = 3). Significance levels versus Api137 are indicated as ** (*p* value < 0.01) and **** (*p* value < 0.0001) by one-way ANOVA with Tukey’s multiple comparison test (GraphPad Prism, 10.4.1).

Two recent reports on Api137 confirmed a previously suggested multimodal mechanism on the bacterial 70S ribosome [26,27], including two binding sites of Api137 at different regions of the 70S ribosome. Ribosomal binding of the fluoroproline-containing Api137 versions was tested by fluorescence polarization on 70S ribosomes isolated from *E. coli* BW25113 in competition to 5(6)-carboxyfluorescein-labeled Api137 (cf-Api137). The dissociation constant (K_d_ = 243.5 ± 15.4 nmol/L) of cf-Api137 and inhibitory constant (K_i_ = 2.63 ± 0.19 nmol/L) of Api137 were similar to published data (Figure 3A and Figure S1) [7,24].

Peptides substituted at Pro5 and Pro9 showed similar K_i_ values ranging from 3.3 to 3.9 nmol/L (Figure 3A and Figure S1), only slightly higher than the K_i_ of Api137. Substitution of Pro11 by 4*S*-Fpr increased the K_i_ approximately 28-fold to 73.39 ± 10.93 nmol/L (Figure 3A and Figure S1), whereas 4*R*-Fpr had no effect (K_i_ = 2.53 ± 0.22 nmol/L), suggesting that the *trans*-conformer is most likely favored in the Api137-ribosome complex. Substitution of Pro13 by 4*S*-Fpr and 4*R*-Fpr increased the K_i_ compared to Api137 to 5.28 ± 0.58 nmol/L and 16.91 ± 1.20 nmol/L, respectively, whereas the corresponding substitutions in position 14 reduced the K_i_ to 1.57 ± 0.12 nmol/L and 1.64 ± 0.14 nmol/L, respectively. The best K_i_ was observed for 4*R*-Fpr in position 16 (0.78 ± 0.22 nmol/L), while the corresponding 4*S*-Fpr-substituted Api137 was worse than Api137 (K_i_ = 4.84 ± 0.78 nmol/L). The unexpected 28- and 8-fold higher K_i_ of 4*S*-Fpr11 and 4*R*-Fpr13 analogs, respectively, than Api137 could be explained by the assumption that the substituted peptide favors one of the two binding sites of cf-Api137.

As all substituted peptides competed with Api137 for its binding sites, their influence on the ribosome activity was studied in an *in vitro* transcription-translation (iTT) assay of superfolder GFP (sfGFP, Figure 3B). Incubation with Api137 (5 µmol/L) reduced sfGFP expression to only 22%, which corresponds to the literature [24]. Most substituted peptides showed a similar reduction ranging from 20 to 35% (Figure 3B). However, incubation with the Api137 analogs 4*R*-Fpr14 and 4*S*-Fpr16 only reduced sfGFP expression to 64% and 63%, respectively, while the corresponding epimers reduced expression to 21% and 28%, respectively. This again shows the importance of the *C*-terminal region of the apidaecins for binding to the ribosomal PET based on the structures of Api137 in complex with the ribosome or trapping the release factor [20,26].

### 2.3. Ribosome Profile Analysis and 50S Subunit Assembly Disruption

**Figure 4:**
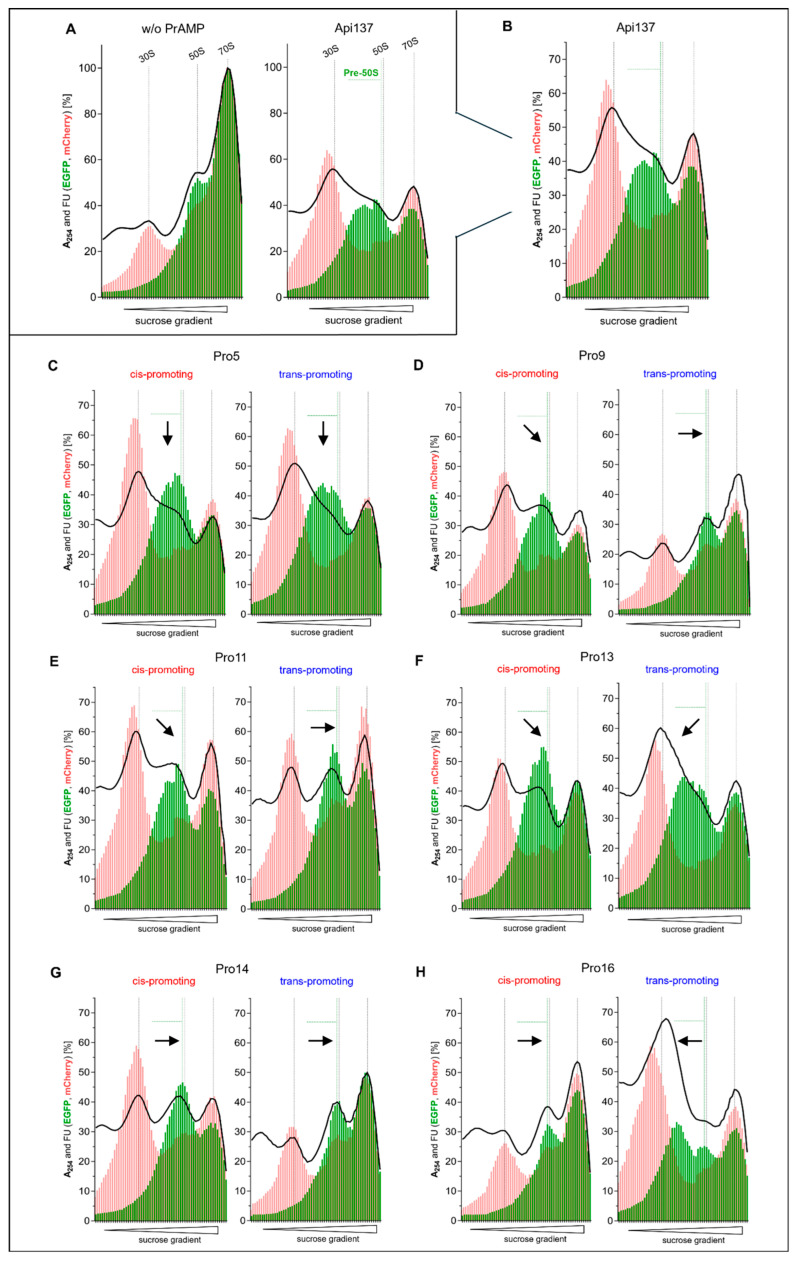
Ribosomal profile analysis of *E. coli* RN31 in the absence ((**A**), *n* = 7) or presence of Api137 ((**A**,**B**), *n* = 7) and its analogs ((**C**–**H**), *n* = 2). *E. coli* RN31 expressing EGFP-bL19 (50S subunit, green bars) and mCherry-bS20 (30S subunit, red bars) were grown in 33% TSB medium and spiked with a PrAMP during the exponential growth phase. After 90 min, cells were lysed and separated by a 5–25% sucrose gradient in TICO buffer. Shown are the absorbance (black line, λ = 254 nm) and fluorescence of EGFP (green bars, λ_ex_ = 485 nm, λ_em_ = 535) and mCherry (red bars, λ_ex_ = 585 nm, λ_em_ = 635 nm) for the fractions relative to the 70S maximum of the control (set to 100%). The dashed black lines shown in all profiles mark the peaks of the 30S and 50S subunits and the 70S ribosome of the control without the addition of a PrAMP ((**A**), **left**). The dashed green lines shown in all profiles (**C**–**H**) mark the pre-50S region relative to the Api137 sample ((**A**), **right** and (**B**)). Shifts in the pre-50S peak maximum relative to Api137 are indicated by black arrows (**C**–**H**). Downward arrows (↓) indicate no shift or no change relative to Api137; diagonal downward arrows (↙ or ↘) indicate a slight shift; and straight arrows (← or →) indicate a strong shift.

Since our previous reports showed the disrupting effect of Api137 on 50S subunit assembly, the impact of the substituted Api137 versions was investigated next [7,27]. The reporter strain RN31 of *E. coli* MC4100 [27] was used because it expresses ribosomal proteins bS20 in the 30S subunit and bL19 in the 50S subunit as fusion proteins with mCherry and EGFP, respectively. The exposed fluorescent proteins on the ribosomal surface do not affect bacterial growth or Api137 susceptibility [27], but allow the specific detection and quantitation of the ribosomal subunits by the corresponding fluorescence [28]. *E. coli* RN31 was incubated with Api137 or an Api137 analog for 90 min, resulting in reduced bacterial growth (Figure 2B). The cell lysate was separated on a sucrose gradient to obtain the ribosomal profiles, which allowed the comparison of the levels of 70S, 50S, pre-50S, and 30S in the bacteria and thus the effect of the studied peptides on ribosome assembly (Figure 4).

The ribosomal profile of *E. coli* RN31 showed the expected large peak of the 70S ribosome and small peaks for the 50S and 30S subunits (Figure 4A). In the presence of Api137, the area of the 70S peak decreased to ~50% and the peak area between the 50S and 30S subunits increased, indicating the increased presence of partially assembled 50S subunits (pre-50S) containing EGFP-tagged bL19. The 30S peak also increased in the mCherry fluorescence and absorbance profiles, indicating an accumulation of correctly assembled 30S subunits, which confirms the results of Lauer et al. [27].

Both peptides substituted at Pro5 showed similar ribosomal profiles, with 4*R*-Fpr5 inducing slightly more 50S precursors than 4*S*-Fpr5 (Figure 4C). Substitution of Pro9 by 4*S*-Fpr reduced the signals for 30S, pre-50, 50S, and 70S compared to Api137. The profile pattern of the 4*R*-Fpr9 analog was similar to the untreated control with no pre-50S peak, only the 70S ribosome peak was reduced to ~45%, similar to the level of Api137 treatment (Figure 4D). Substitution of Pro11 in the middle of the peptide chain showed weaker effects on 50S assembly than Api137, with no pre-50S peak visible for the 4*R*-Fpr11 analog and a weaker pre-50S peak observed for the 4*S*-Fpr11 analog (Figure 4E). When Pro13 was substituted, the 70S peak areas of both analogs were similarly slightly reduced relative to Api137, whereas the 30S, 50S, and pre-50S regions were different between the two analogs (Figure 4F). The 4*S*-Fpr substitution resulted in a lower intensity for the 30S peak and showed a narrow, more intense 50S peak and a weak pre-50S peak, while the 4*R*-Fpr13 analog showed a high content of pre-50S and 30S comparable to Api137 (Figure 4F). The corresponding Pro14 analogs reduced the 70S peak without the accumulation of pre-50S (Figure 4G), while the 4*R*-Fpr14 profile showed similarities to the untreated control. The strongest effects relative to Api137 were observed for the analogs substituted at Pro16 (Figure 4H). The 4*S*-Fpr analog showed a ribosomal profile similar to the control in the absence of PrAMP except for the weaker 70S signal, which was still more intense than in Api137 and all other substituted Api137 peptides. In contrast, the ribosomal profile obtained for Api137 substituted with 4*R*-Fpr in position 16 showed 70S and 30S peaks with a similar intensity to that of Api137, whereas the 50S signal was strongly reduced and a well-separated pre-50S signal appeared with higher intensity than the 50S signal, indicating a high content of small 50S precursors, suggesting a strong inhibition of the 50S subunit assembly. While the overall intensity of the green fluorescence from EGFP is comparably low to the ribosomal profiles observed for the other peptides, the absorbance was significantly higher in the region between the 30S and 50S subunits, suggesting the presence of small 50S precursors that lack EGFP-tacked bL19.

Substitution of proline residues with 4*S*-Fpr or 4*R*-Fpr at *N*-terminal position Pro5 and mid-chain position Pro13 did not alter ribosome 50S assembly and 70S formation relative to Api137. In contrast, mid-chain positions Pro9 and Pro11, and particularly substitutions of Pro14 and Pro16, showed stronger effects. 4*R*-Fpr14 and 4*S*-Fpr16 analogs largely abolished the effects of Api137 on the ribosomal profile, resulting in a profile similar to the control except for a weaker 70S peak (Figure 4G,H). The 4*S*-Fpr14 analog resulted in a disassembled 70S without increased pre-50S accumulation, whereas the 4*R*-Fpr16 analog showed the strongest effect on pre-50S accumulation. Interestingly, substitution of Pro14 with either 4*S*-Fpr or 4*R*-Fpr slightly improved the MICs and K_i_ values compared to Api137, whereas the 4*R*-Fpr analog showed only a weak inhibition of sfGFP production in the iTT assay (Figure 3B). When Pro16 was substituted, the 4*R*-Fpr analog showed a similar inhibition of sfGFP production to that of Api137, whereas the 4*S*-Fpr analog had a much weaker inhibitory effect, which corresponded well to the MICs and growth inhibition of both analogs (Figure 2). This, together with the three-times-better K_i_ values and the larger area of the pre-50S peak precursor relative to Api137, suggests that the *trans*-promoting 4*R*-Fpr favors a conformation that allows the subsequent Arg17, which is very important for the antimicrobial activity of Api137 [20], to bind better than in the Pro-Arg motif of Api137. This is further supported by the opposite effects observed for the 4*S*-Fpr16 analog.

### 2.4. Further Analysis of the C-Terminal Proline Substitutions

The strong influence of the substitutions of Pro14 and Pro16 in different experiments raised the question of whether they were due to favorable or unfavorable *C*-terminal peptide conformers or altered the mode of action. Thus, we studied the growth of *E. coli* at different peptide concentrations (Supplementary Figure S2) and adjusted the peptide concentrations to obtain the growth inhibition of all four substituted peptides closer to that of Api137 (8 µg/mL, 70% inhibition), namely 16 µg/mL for the 4*S*-Fpr14 analog (64% inhibition), 32 µg/mL for the 4*R*-Fpr14 (37% inhibition) and 4*S*-Fpr16 analogs (33% inhibition), and 4 µg/mL for the 4*R*-Fpr16 analog (70% inhibition) (Supplementary Figure S3A).

When *E. coli* was incubated with the adjusted peptide concentration of the 4*S*-Fpr14 analog, a pre-50S peak was observed (Supplementary Figure S3B) that was not detected at the two-fold-lower peptide concentration (Figure 4G). This result supports our hypothesis that the peptide conformer is crucial for inhibition of 50S assembly, and thus a higher peptide concentration is required to obtain the effect for Api137 analogs containing a lower proportion of the preferred conformer. Accordingly, the lower concentration of the 4*R*-Fpr16 analog reduced the area of the pre-50S peak (Supplementary Figure S3C) approximately to the same level as observed for Api137 at a two-times-higher concentration (Figure 4B). The quadrupled concentrations of the 4*R*-Fpr14 and 4*S*-Fpr16 analogs resulted in similar ribosomal profiles to those seen for their lower concentrations and close to those of untreated *E. coli* cells, which is consistent with the lower inhibitory effect on *E. coli* growth in 90 min. However, these conclusions rely on the assumption that the intracellular peptide concentrations of the PrAMPs studied correlate with the medium concentration.

**Figure 5:**
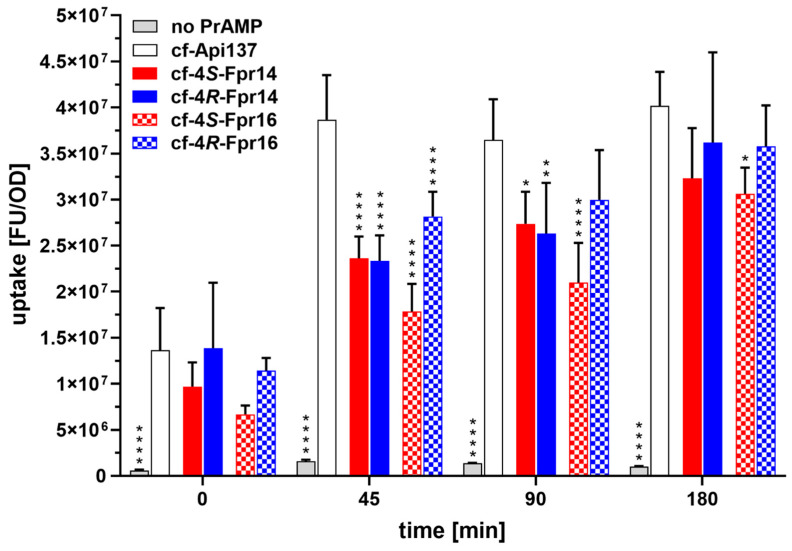
Bacterial uptake of cf-Api137 and four selected analogs. Cf-labeled PrAMPs were spiked into exponentially growing *E. coli* MC4100 and incubated in 33% TSB medium. Shown are the ratios of fluorescence (λ_ex_ = 485 nm, λ_em_ = 535 nm) representing the labeled peptide to OD_600_ representing the cell number in bacterial cell pellets after incubation periods of 0, 45, 90, and 180 min with no peptide (gray), Api137 (white), and 4*S*-Fpr (red) or 4*R*-Fpr (blue) at positions 14 (solid bars) or 16 (checkered bars). Error bars represent the mean of three individual experiments (*n* = 3). Significance levels versus cf-Api137 are indicated as * (*p* value < 0.1), ** (*p* value < 0.01), *** (*p* value < 0.001), and **** (*p* value < 0.0001) by one-way ANOVA with Tukey’s multiple comparison test (GraphPad Prism, 10.4.1).

The cellular uptake of analogs substituted at Pro14 or Pro16 was only ~46% to 73% of that of cf-Api137 during the first 45 min (Figure 5). Both isomers of the Fpr14 analog were taken up at similar rates (60% to 61%), while the 4*R*-Fpr16 analog was taken up by ~73% and the 4*S*-Fpr16 analog was taken up by ~46%. The intracellular peptide concentration of cf-Api137 remained constant until 180 min, while the uptake of the cf-Fpr analogs continued to reach levels of ~76% to ~90% relative to Api137 after 180 min. The labeled 4*S*-Fpr16 analog showed the slowest uptake rate consistently. It is unclear whether the uptake is slower due to slower diffusion through the outer bacterial membrane or less efficient SbmA transport. However, the changes in the activity of the analogs appear to depend on either the uptake kinetics within the first 45 min or ribosome binding.

## 3. Discussion

The antibacterial properties of apidaecin-like peptides have been extensively studied *in vitro* and *in vivo*, including structural modifications to improve activity against human pathogens and to stabilize the sequence against proteolytic degradation. The best studied apidaecin-like peptides are Api88 and Api137, which have been extensively investigated in murine infection models to evaluate their pharmaceutical potential and by cryo-EM to reveal their binding sites on the bacterial ribosome [11,25,38,39]. Although Api88 and Api137 were reported more than 10 years ago, subsequent research efforts based on screening techniques have not been able to further improve their antibacterial activity or find other apidaecin-derived peptides with higher activities, suggesting that near-optimal activity has been achieved. However, structural data using cryo-EM have revealed the binding sites of Api88 and Api137 on the *E. coli* ribosome, which may provide a new opportunity to optimize the peptide for better target binding [20,26,27]. Although better binders will not necessarily show higher antibacterial activity, as bacterial uptake and off-target binding are not considered, they may provide new lead structures to be further optimized for improved uptake rates due to *C*-terminal amidation (i.e., Api88 versus Api137) or more favorable interactions with the SbmA transporter, as suggested by A. Mankin to explain the higher antibacterial activity of Fva1 compared to the highly homologous apidaecin 1b despite their identical interactions with the ribosome [36,40]. Mankin’s group also postulated that apidaecin-type PrAMPs could act as *cis* or *trans* conformers in the PET of the 70S ribosome [36]. Considering the peptide structures of Api88 and Api137 in complex with the *E. coli* 70S ribosome, which indicate a *trans*-conformer of all Xaa-Pro bonds in Api137 [20,26], we hypothesized that substitution of Pro residues by 4*R*-Fpr should favor the *trans*-conformer and promote binding, whereas 4*S*-Fpr substitutions would have opposite effects. Moreover, the peptide structure adopted by the peptide bound to the PTC binding site indicated the importance of the proline-rich region, which mostly retained its conformation in molecular dynamics simulations [26]. This region, which includes the five *C*-terminal residues of Api137 (PHPRL) with Pro14 and Pro16, has been proposed as the pharmacophore unit [21,30,37].

Because the structures and net charges of the 4*S*- and 4*R*-Fpr analogs are very similar to those of Api137 and the absence of lytic effects against *E*. *coli*, the cytotoxicity profile should resemble that of Api137, which is virtually non-toxic to eukaryotic cell lines [21] and is well tolerated in mice [11,38,39]. Since Api137 binds to 70S ribosome preparations from other Gram-negative bacteria as well as to *E. coli* ribosomes [41] and is highly active against these bacteria [21,41], the results shown here for *E. coli* are likely valid for other Gram-negative bacteria as well.

The most compelling results were obtained for Pro16, where the *cis*/*trans* orientation of the Xaa-Pro bond in either *cis* or *trans* should have a major impact on the positioning of the subsequent residues Arg17 and Leu18, which interact at the PTC binding site with the 23S rRNA, the release factor, and the deacylated peptidyl-tRNA [20]. In addition, the *C*-terminal sequence plays an important role in the PET exit binding site by colliding with the early assembly protein uL22 in 50S precursors, preventing proper assembly of the 50S subunit, and leading to non-functional dead-end particles [27]. As hypothesized, the analog containing *trans*-promoting 4*R*-Fpr in position 16 bound more strongly to the ribosome than Api137, reduced bacterial growth more strongly than Api137, and inhibited 50S subunit assembly more strongly than Api137, resulting in even smaller precursors. Most likely, 4*R*-Fpr in position 16 induces a conformation that positions Arg17 and the *C*-terminus in a more favorable position to sterically hinder the binding of uL22 in the early assembly steps. Interestingly, the stronger effects on the ribosome did not improve the MIC compared to Api137. Consistent with our hypothesis, the *cis*-promoting 4*S*-Fpr16 analog did not inhibit 50S subunit assembly and only slightly reduced *in vitro* translation and bacterial growth. This peptide also had the highest MIC, which corresponds to the activity reported for Api137 analogs containing *cis*- or *trans*-4-hydroxy-l-proline in position Pro16 [21]. Furthermore, the uptake of the 4*S*-Fpr16 analog was less efficient than that of Api137, suggesting that either the *cis* conformer is less able to penetrate the outer membrane or is less efficiently transported by SbmA, although this transporter is known to transport peptides and proteins with different tertiary structures [8,42]. Similar differences in uptake of apidaecin analogs with slightly altered structures have been reported previously [24], although it remains unclear which step of uptake is affected. However, the data clearly indicated that the *C*-terminal pharmacophore unit is also important for bacterial uptake.

The effect of small-molecule antibiotics on ribosome assembly has been the subject of considerable debate, with some research groups interpreting it as a direct effect on the assembly and others suggesting a sequential effect due to irregular translation [28,43,44,45,46]. Recently, we provided evidence for a direct effect of Api137 on ribosomal subunit assembly by cryo-EM, which clearly showed an imbalance in 70S assembly and structural differences in pre-50S particles, resulting in mostly misassembled dead-end particles [27]. This study also highlighted the importance of the *C*-terminal region in disrupting 50S subunit assembly, as Api88, the *C*-terminally amidated version of Api137, did not significantly affect assembly. Interestingly, the 4*S*-Fpr16 analog also did not affect the assembly, whereas the 4*R*-Fpr16 analog showed an even stronger inhibition of the 50S subunit assembly, shifting the peak to even smaller 50S precursors than Api137.

Strikingly, inhibition of *in vitro* translation and 50S precursor stalling did not correlate, suggesting that these are two independent mechanisms requiring different structures of the apidaecin analogs. This is most evident for the 4*S*-Fpr14 analog, which showed the strongest inhibitory effect on sfGFP production and a similar ribosomal profile to Api137 except for the missing pre-50S peak. Almost identical effects were observed for the 4*S*/*R*-Fpr9 analogs. Therefore, the interaction during 50S assembly is likely a consequence of the preferred conformation of the peptide. The preferred conformation of the *C*-terminal sequence determines whether the peptide interferes with later assembling proteins and stalls assembly at the pre-50S level or inhibits protein translation on the functional 70S ribosome without affecting the assembly process. Our hypothesis of bimodal activity targeting protein translation on the 70S ribosome or the 50S assembly is further supported by the observed dose-dependent effects: At low concentrations, the preferred peptide conformation inhibits one process, whereas at a higher peptide concentration, both effects can be observed, which is consistent with the minimal concentration of the corresponding peptide conformer required to observe visible effects on *in vitro* translation or the ribosome profile.

While these data are compelling, we would like to stress that this study focused mostly on the bacterial ribosome as the primary target of apidaecins, including the known apidaecin binding sites. In this respect, it is limited to the ribosome as the target and does not address the exact binding site, contact area, or the cellular environment, which is much more complex. The bacterial uptake depends on the physicochemical properties of a peptide to diffuse through the outer membrane, enter the periplasm, bind to the SbmA transporter, and pass the inner membrane. While this two-step mechanism is widely accepted, the specific residues and sequence that favor cellular uptake remain unclear. For example, Api88 is taken up much faster and reaches higher intracellular concentrations than Api137 in *E. coli*, although both peptides are similarly active against different *E. coli* strains. Given the faster uptake of Api88 relative to Api137, it will be interesting to see if *C*-terminal amidation will also accelerate the uptake of the corresponding Fpr14 and Fpr16 analogs. Other important aspects contributing to antibacterial activity include interactions with other intracellular structures or biopolymers (e.g., off-target binding with the heat shock protein DnaK), and degradation by periplasmic or cytosolic proteases. *In vitro* studies considering target binding, bacterial uptake, and inhibition of 50S subunit assembly as well as structural studies using cryo-EM, computational docking studies, and molecular dynamics simulations can further guide the rational optimization of promising 4*S*-Fpr11- and 4*R*-Fpr16-substituted Api137 and Api88 analogs. However, such studies cannot address all *in vivo* effects, which may differ significantly for substituted analogs. Overall, we cannot explain why the 4*R*-Fpr14 analog is more active against *E. coli* than Api137 despite its lower inhibition of the *in vitro* translation of sfGFP and lack of effect on the ribosomal profile. Similarly, the high activity of the 4*S*-Fpr11 analog contrasts with its 30-fold-higher Ki, lower uptake, and equal inhibition of 50S subunit assembly compared to Api137. These findings could be related to a weaker off-target binding or suggest a novel mechanism that differs from or has not been identified for Api137. A mechanism independent of translation stalling was already suggested in two previous reports for *C*-terminally modified versions of Api137, i.e., Api88 and Api805, which showed almost no RF-dependent inhibition of sfGFP production [24,26]. This was further supported for Api88, which showed a more flexible binding to the ribosome as well as an additional binding site [26], raising the possibility that the 4*R*-Fpr14 analog may have a different ribosome binding behavior or another unknown mode of action.

## 4. Materials and Methods

### 4.1. Materials and Chemicals

Reagents used are listed in the Supplementary File S1. All 9-fluorenylmethyloxycarbonyl- (Fmoc-) protected amino acid derivatives used for peptide synthesis were obtained from Orpegen Pharma GmbH (Heidelberg, Germany) and Iris Biotech (Marktredwitz, Germany). (2*S*,4*S*)-Fmoc-l-Pro(4-F)-OH and (2*S*,4*R*)-Fmoc-l-Pro(4-F)-OH were either synthesized in-house [47,48] or obtained from Iris Biotech (Marktredwitz, Germany). Water (resistance  ≥  18 mΩ, total organic content  <  1 ppb) was purified in-house using a PureLab Ultra Analytic system (ELGA Lab Water, Celle, Germany).

### 4.2. Peptide Synthesis

Peptides were synthesized on solid phase using a multiple synthesizer (SYRO2000, MultiSynTech GmbH, Witten, Germany), Fmoc/*^t^*Bu-chemistry, in situ activation with DIC in the presence of HOBt, and Wang resins to obtain *C*-terminal acids, respectively (Table 1). The *N*-termini of Api137 and related sequences were tetramethylguanidinated with HBTU in the presence of NMM. In these peptides, Orn-1 was incorporated as Fmoc-Orn(Mtt)-OH, which was selectively deprotected with 2% (*v*/*v*) TFA and 2.5% (*v*/*v*) TIS in DCM after *N*-terminal guanidination. 5(6)-Carboxyfluorescein (cf) was coupled with HBTU in the presence of DIPEA to either an unprotected *N*-terminus or to the δ-amino group of Orn-1 for *N*-terminally tetramethylguanidinated sequences. Fmoc-Fpr-OH (4 eq) was manually coupled by DIC activation (8 eq) for two hours and incubated for another two hours after the addition of fresh DIC (4 eq). Peptides were cleaved with TFA containing a 12.5% (*v*/*v*) scavenger mixture (ethanedithiol, *m*-cresol, thioanisole, and water; 1:2:2:2 (by vol)) and precipitated with cold diethyl ether. Peptides were purified on an Äkta Purifier 10 using a Jupiter C_18_-column (ID 10 mm or 21.2 mm) with an aqueous acetonitrile gradient in the presence of 0.1% TFA as an ion pair reagent. Peptide purity was analyzed by RP-HPLC on a Jupiter C_18_-column (ID 4.6 mm or 2 mm) and the absorbance was recorded at 214 nm. Molecular weights were confirmed by matrix-assisted laser desorption/ionization time-of-flight mass spectrometry (MALDI-TOF-MS; 5800 Proteomic Analyzer; AB Sciex, Darmstadt, Germany) or by ESI-MS (Esquire HCT; Bruker Daltonics, Bremen, Germany) (Supplementary File S2).

### 4.3. Ribosome Preparation

*E. coli* BW25113 were cultivated in LB medium and harvested by centrifugation (5000× *g*, 15 min, 4 °C) at an OD_600_ of 3–4. Cell pellets were washed in ribosome preparation buffer (20 mmol/L HEPES-KOH, 6 mmol/L MgCl_2_, 30 mmol/L NH_4_Cl, pH 7.6, 4 °C), centrifuged (4100× *g*, 30 min, 4 °C), and resuspended in ribosome preparation buffer (0.5 g/L) containing freshly added 2-mercaptoethanol (4 mmol/L) and lysozyme. Cells were incubated on ice for 30 min and disrupted in six cycles using a FastPrep-24^TM^ 5G instrument (MP Biomedicals, Eschwege, Germany) with the BigPrep 50 mL setting (40 s, 4.0 m/s), interrupted by one minute of incubation on ice between the cycles. After a centrifugation step (1620× *g*, 5 min, 4 °C), DNase I (5 U/mL) was added to the supernatant and incubated for 1 h on ice. Cell debris was removed by centrifugation at 16,000× *g* (30 min, 4 °C) and twice at 32,000× *g* (60 min, 4 °C). Crude ribosomes were obtained by centrifugation at 165,000× *g* (17 h, 4 °C). The pellet was washed and resuspended in ribosome preparation buffer to obtain the 70S ribosome extract.

### 4.4. Fluorescence Polarization

K_d_ values were measured using 70S ribosome extract and cf-labeled peptides in ribosome preparation buffer. Briefly, black 384-well plates (flat bottom, PP, 781209, Greiner Bio-One GmbH, Frickenhausen, Germany) were blocked with casein (0.5%, *w*/*v*) in PBS containing 0.05% (*v*/*v*) Tween 20 (PBS-T) at 4 °C overnight and washed three times with PBS-T. A 2-fold dilution series (22 steps) of 70S ribosome extract (10 μL per well) in ribosome preparation buffer was performed in a black 384-well plate. The cf-labeled peptide was dissolved in ribosome preparation buffer (10 μL, 40 nmol/L), added to each well, centrifuged (500× *g*, 2 min), and incubated at 28 ± 1 °C for 90 min. Fluorescence polarization was determined on a PARADIGM^TM^ microplate reader (Beckman Coulter, Krefeld, Germany) in the top reading position (λ_ex_ = 485 nm, λ_em_ = 535 nm). Data were fitted to a nonlinear dose–response logistic transition equation [y = y0 + a/(1 + (x/x0)b)] using the Levenberg–Marquardt algorithm, with K_d_ represented by the x0 coefficients (GraphPad Prism for Windows v. 5.02, San Diego, CA, USA).

Inhibition constants (K_i_) were measured for the 70S ribosome extract using cf-labeled and unlabeled peptides in ribosome preparation buffer. Unlabeled peptides (20 µL, 300 µmol/L) were added to ribosome preparation buffer in a 2-fold serial dilution series of 21 steps in a black 384-well plate. A ribosome solution adjusted to a ribosome concentration corresponding to ~90% of the upper K_d_ plateau obtained for the cf-labeled peptide of interest was added to each well (10 µL). After incubation (28 °C ± 1 °C, 90 min), the cf-labeled peptide (10 µL, 80 nmol/L) was added and incubated (28 °C ± 1 °C, 90 min). Fluorescence polarization was determined on a PARADIGM^TM^ microplate reader in the top reading position (λ_ex_ = 485 nm, λ_em_ = 535 nm). Data were fitted to a nonlinear dose–response logistic transition equation [y = y0 + a/(1 + (x/x0)b)] using the Levenberg–Marquardt algorithm, with half-maximal inhibition constants (IC_50_) represented by the x0 coefficients (GraphPad Prism for Windows v. 5.02, San Diego, CA, USA). K_i_ values were calculated as described by Mathias and Jung [49].

### 4.5. Antibacterial Activity

Minimum inhibitory concentrations were determined for *E. coli* RN31 grown in 33% TSB (37 °C, 200 rpm) with a starting OD_600_ of 0.05. After four hours, the cell count was adjusted to 1.5 × 10^7^ bacteria/mL. In a transparent 96-well plate, the peptides were diluted in a two-fold dilution series in 33% TSB over 10 dilution steps, and *E. coli* was added to obtain a final cell count of 7.5 × 10^6^ bacteria/mL and incubated at 37 °C overnight. OD_600_ was determined using a PARADIGM^TM^ microplate reader (Beckman Coulter, Salzburg, Austria) before and after cultivation. The MIC was defined as the lowest peptide concentration preventing visible bacterial growth.

### 4.6. In Vitro Transcription/Translation (iTT) Assay

The sfGFP expression analysis relied on the NEB PureExpress Delta RF123 Kit (New England Biolabs, Frankfurt, Germany). The DNA template encoding sfGFP with a UAG stop codon was amplified from the pY71sfGFP vector by PCR using the primers sfGFP fwd (TAATACGACTCACTATAGGG) and sfGFP rev (CATGAAGCTTATTTTTCGAACTGCGGAT). The release factor RF1 of the kit was diluted 50-fold (*v*/*v*) in 1× Pure System Buffer (PSB, 1 mol/L magnesium acetate, 0.5 mol/L K_3_PO_4_ (pH 7.3), 1 mol/L potassium glutamate, 1 mol/L NH_4_Cl, 0.5 mol/L CaCl_2_, 1 mol/L spermidine, 0.1 mol/L putrescine, 0.1 mol/L DTT). sfGFP expression was performed in a freshly prepared mixture of kit solution A (2 µL), kit solution B (1.5 µL), 50-fold diluted RF1 (0.5 µL), sfGFP template (0.1325 pmol, 35 ng) or water as control (0.25 µL), PSB (0.25 µL), and a PrAMP (50 or 0 µmol/L) dissolved in water (0.5 µL). The reaction mixture was transferred to a black 384-well plate (flat bottom, PS, 784900, Greiner Bio-One GmbH), covered with a lid, and incubated (37 °C, 2 h) in a microplate reader (Gemini EM, Molecular Devices, San Jose, CA, USA). Fluorescence was recorded every 10 min (λ_ex_ = 485 nm, λ_em_ = 535 nm).

### 4.7. Cultivation of E. coli RN31 for Ribosomal Profile Analysis

*E. coli* RN31 was grown in 33% TSB (37 °C, 200 rpm) from a starting OD_600_ of 0.05 to an OD_600_ of 0.2, measured on a NanoPhotometer^®^ (NP80; Implen; Westlake Village, CA, USA), and divided into subcultures that were inoculated with a peptide (8 µg/µL) before being cultured for 90 min (37 °C, 200 rpm). The following steps were performed on ice. Cells were harvested by centrifugation (4000× *g*, 4 °C, 25 min), washed with TICO buffer (20 mmol/L Hepes-KOH, pH 7.6, 6 mmol/L MgAc_2_, 30 mmol/L KAc), centrifuged (4000× *g*, 25 min, 4 °C), and stored at −80 °C. The cell pellet was suspended in TICO buffer (500 µL) and mixed with silica beads (zirconium glass pellets, 100 µm diameter; Carl Roth; Karlsruhe, Germany) to lyse the cells on a FastPrep-24^TM^ 5G (MP Biomedicals, Solon, OH, USA) using three disruption cycles (30 s, 6.0 m/s) interrupted by 1 min of incubation on ice after each cycle. Cell debris was removed by centrifugation (5000× *g*, 4 °C, 5 min), the supernatant was transferred to a new reaction tube, centrifuged (20,000× *g*, 4 °C, 20 min), the absorbance was recorded at 260 nm on a NanoPhotometer^®^ NP80, and the sample was stored at −80 °C.

### 4.8. Ribosomal Profile Analysis

A 5% (*w*/*v*) sucrose solution (6.5 mL) in TICO buffer containing 2-mercaptoethanol (4 mmol/L) was poured into an open-top centrifuge tube (13.2 mL, 14 × 89 mm, Beckman Coulter) and bottomed with a 25% (*w*/*v*) sucrose solution (6.5 mL) in TICO buffer containing 2-mercaptoethanol (4 mmol/L) using a cannula. The tube was sealed with parafilm and placed horizontally at 4 °C for 3 h and vertically at 4 °C for 30 min. A portion of the upper layer (550 µL) was replaced by lysate (500 µL, A_260_ = 30) and centrifuged using a swing-out rotor (SW 41 Ti, 52,500× *g*, 4 °C, 19 h) without a brake. The contents were transferred from bottom-up to obtain equal volumes in all wells of a black 96-well plate with a flat bottom (96 Well PP black Greiner Bio-One GmbH, Frickenhausen, Germany) using the Äkta Purifier 10 system, and absorbance was recorded at a wavelength of 254 nm. Fluorescence was measured in the plate using the PARADIGM at excitation (λ_ex_) and emission (λ_em_) wavelengths of 485 nm and 535 nm for eGFP and 585 nm and 635 nm, respectively, for mCherry and normalized to the 70S ribosome peak (set to 100%).

### 4.9. Cellular Uptake

Bacterial uptake of the peptides was investigated in *E. coli* MC4100 using a recently published protocol [24]. Briefly, bacteria were grown in TSB (33%) overnight (37 °C, 200 rpm) to inoculate fresh medium with an initial OD_600_ of 0.3. When the OD_600_ was 1 to 1.1, the culture was split and a cf-labeled peptide was added to a final concentration of 23 µmol/L. After incubation in the dark for 0, 45, 90, and 180 min, an aliquot (600 µL) was taken, split in half, and immediately placed on ice. An aliquot of each sample (150 µL) was used to determine the OD_600_ value of the bacterial culture (transparent 96-well plate), and 100 µL of each was transferred to a black 96-well plate for fluorescence determination. The remaining samples were centrifuged (10,000× *g*, 3 min), and aliquots of the supernatant (100 µL each) were transferred to a black 96-well plate. The pellet was resuspended in PBS (300 µL), centrifuged (10,000× *g*, 3 min), and the pellet was washed twice with PBS (100 µL). The resuspension and washing were repeated once. The pellet was transferred to a black 96-well plate, and OD_600_ and fluorescence were recorded as described above. The fluorescence was normalized to OD_600_. Three individual experiments were performed, including two technical replicates each.

### 4.10. Bacterial Growth Rate

*E. coli* RN31 grown in TSB (33%) overnight (37 °C, 200 rpm) was used to inoculate fresh medium. When the OD_600_ was ~0.2, 95 µL were transferred to a transparent 96-well plate, and 5 µL of a two-fold dilution series of PrAMPs were added. The 96-well plate was transferred directly to a PARADIGM^TM^ microplate reader (Beckman Coulter, Krefeld, Germany), incubated at 37 °C without shaking for 24 h, and OD_600_ was recorded every 15 min with 5 s medium strength orbital shaking prior to each measurement. Two biological replicates were measured.

## 5. Conclusions

The interaction and mode of action of the unordered flexible peptide chains of Api137 and its analogs depend on the content of *cis* and *trans* conformers at the Xaa-Pro bonds, which was observed by substituting Pro9 and Pro11 in the mid-chain position and especially Pro14 and Pro16 in the *C*-terminal pharmacophore unit by 4*S*- or 4*R*-Fpr, which stabilize the *cis* or *trans* conformers, respectively (Table 2).

This shift in conformer ratios may favor one of the dual mode mechanisms reported for Api137 or provide a tool to identify undiscovered mechanisms, as suggested by a third binding site of Api88, with currently unknown effects on ribosome activity. From a pharmacological point of view, the potent inhibition of ribosome assembly observed for the 4*R*-Fpr16 analog may provide a novel, clinically unexploited mechanism to pursue for future antibiotic development.

## Figures and Tables

**Table 1:** Sequences of all synthesized peptides. Gu: N,N,N′,N′-tetramethylguanidino; O: ornithine; red P: 4*S*-Fpr (*cis*-promoting); and blue P: 4*R*-Fpr (*trans*-promoting).

PrAMP	Substitution (Position)	Sequence
Api137	None	gu-ONNR**P**VYI**P**R**P**R**PP**H**P**RL-OH
Api839	4*S*-Fpr (5)	gu-ONNR**P**VYIPRPRPPHPRL-OH
Api840	4*R*-Fpr (5)	gu-ONNR**P**VYIPRPRPPHPRL-OH
Api845	4*S*-Fpr (9)	gu-ONNRPVYI**P**RPRPPHPRL-OH
Api846	4*R*-Fpr (9)	gu-ONNRPVYI**P**RPRPPHPRL-OH
Api841	4*S*-Fpr (11)	gu-ONNRPVYIPR**P**RPPHPRL-OH
Api842	4*R*-Fpr (11)	gu-ONNRPVYIPR**P**RPPHPRL-OH
Api843	4*S*-Fpr (13)	gu-ONNRPVYIPRPR**P**PHPRL-OH
Api844	4*R*-Fpr (13)	gu-ONNRPVYIPRPR**P**PHPRL-OH
Api847	4*S*-Fpr (14)	gu-ONNRPVYIPRPRP**P**HPRL-OH
Api848	4*R*-Fpr (14)	gu-ONNRPVYIPRPRP**P**HPRL-OH
Api849	4*S*-Fpr (16)	gu-ONNRPVYIPRPRPPH**P**RL-OH
Api850	4*R*-Fpr (16)	gu-ONNRPVYIPRPRPPH**P**RL-OH

**Table 2:** Comparison of antimicrobial activity against *E. coli* RN31 (MIC), 70S ribosome binding in competition with cf-Api137 (K_i_), effect on sfGFP expression in an *in vitro* transcription-translation assay (iTT), and formation of pre-50S states in a ribosome profile analysis (RPA) of Api137 and all 4*S*- and 4*R*-fluoro-l-proline (Fpr) substituted Api137 analogs used in the current study. Marked values indicate significant improvements (green) or deterioration (red) of the analogs compared to Api137.

	Substitution (*Promoting Conformer*)	MIC [µg/mL]	Ki [nM]	Residual iTT [%]	RPA [pre-50S Formation]
Api137	None	4	2.6 ± 0.2	22 ± 6.2	yes
Pro5	4*S*-Fpr (*cis*)	4	3.3 ± 0.5	23 ± 0.2	yes
	4*R*-Fpr (*trans*)	4	3.3 ± 0.3	35 ± 0.1	yes
Pro9	4*S*-Fpr (*cis*)	2	3.9 ± 0.4	26 ± 5.7	yes
	4*R*-Fpr (*trans*)	4	3.7 ± 0.4	33 ± 3.5	no
Pro11	4*S*-Fpr (*cis*)	1	73.4 ± 10.9	32 ± 0.9	yes
	4*R*-Fpr (*trans*)	4	2.5 ± 0.2	31 ± 0.7	reduced
Pro13	4*S*-Fpr (*cis*)	4	5.3 ± 0.6	28 ± 3.2	yes
	4*R*-Fpr (*trans*)	2	16.9 ± 1.2	28 ± 1.2	yes
Pro14	4*S*-Fpr (*cis*)	2	1.6 ± 0.1	21 ± 0.2	reduced
	4*R*-Fpr (*trans*)	2	1.6± 0.1	64 ± 4.6	no
Pro16	4*S*-Fpr (*cis*)	16	4.8 ± 0.8	63 ± 8.9	no
	4*R*-Fpr (*trans*)	4	0.8 ± 0.2	28 ± 0.7	yes

## Data Availability

The data presented in this study are available on request from the corresponding author.

## Supplementary Materials

The following supporting information can be downloaded at: https://www.mdpi.com/article/10.3390/antibiotics14060566/s1. Figure S1: Inhibition of cf-Api137 competing with unlabeled Api137 and 4S-Fpr and 4R-Fpr analogs for binding to the 70S ribosome; Figure S2: Growth curves of E. coli RN31 cultures incubated without and with Api137 and its analogs substituted with 4S-Fpr or 4R-Fpr at positions 14 or 16 at different concentrations; Figure S3: Growth rates of different PrAMPs and ribosomal profiles of E. coli RN31 incubated with Api137 analogs substituted with 4S- or 4*R*-Fpr at positions 14 or 16; File S1: Material and Chemicals—Reagents; File S2: Mass spectra of the 4*S*-Fpr and 4*R*-Fpr substituted Api137 analogs.
