# Supplementary material for: Substitution of Proline Residues by 4-Fluoro-l-Proline Affects the Mechanism of the Proline-Rich Antimicrobial Peptide Api137
Source: Antibiotics (Basel). 2025 May 31;14(6):566. doi: 10.3390/antibiotics14060566 (PMC12189828; doi:10.3390/antibiotics14060566)

Supplementary Materials

Substitution of Proline Residues by 4-Fluoro-L-Proline Affect the Mechanism of the Proline-Rich Antimicrobial Peptide Api137

Maren Reepmeyer, Andor Krizsan, Alexandra Brakel, Lisa Kolano, Jakob Gasse, Benjamin W. Husselbee, Andrea J. Robinson and Ralf Hoffmann

Figure S1: Inhibition of cf-Api137 competing with unlabeled Api137 and 4*S*-Fpr and 4*R*-Fpr analogs for binding to the 70S ribosome S2

Figure S2: Growth curves of *E. coli* RN31 cultures incubated without and with Api137 and its analogs substituted with 4*S*-Fpr or 4*R*-Fpr at positions 14 or 16 at different concentrations S3

Figure S3: Growth rates of different PrAMPs and ribosomal profiles of *E. coli* RN31 incubated with Api137 analogs substituted with 4*S*- or 4*R*-Fpr at positions 14 or 16 .... S4-5

File S1: Material and Chemicals - Reagents S6

File S2: Mass spectra of the 4*S*-Fpr and 4*R*-Fpr substituted Api137 analogs............ S7-11

A

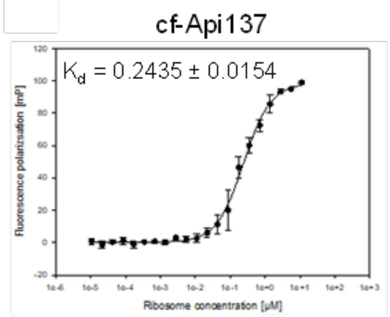

B

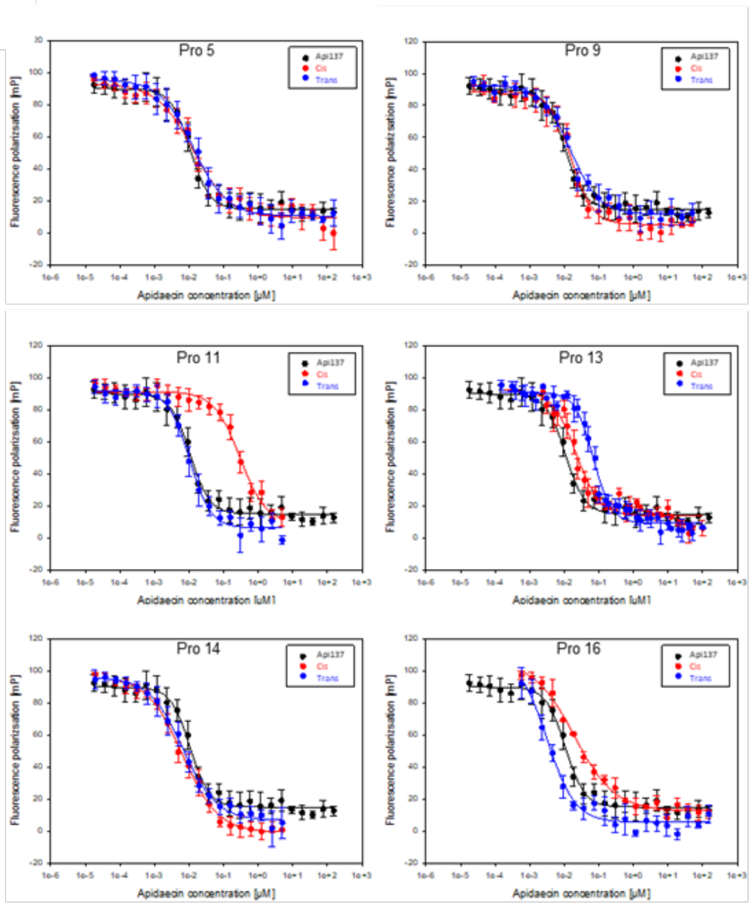

C

Peptide	IC_{50} [nM]	K_i [nM]
Api137	10.7 ± 0.8	2.63 ± 0.19
4S-Fpr5	13.5 ± 2.1	3.31 ± 0.51
4R-Fpr5	13.4 ± 1.4	3.29 ± 0.34
4S-Fpr9	15.8 ± 1.7	3.88 ± 0.41
4R-Fpr9	15 ± 1.5	3.68 ± 0.36
4S-Fpr11	298.7 ± 44.5	73.39 ± 10.93
4R-Fpr11	10.3 ± 0.9	2.53 ± 0.22
4S-Fpr13	21.5 ± 2.4	5.28 ± 0.58
4R-Fpr13	68.8 ± 4.9	16.91 ± 1.20
4S-Fpr14	6,4 ± 0.5	1.57 ± 0.12
4R-Fpr14	6,7 ± 0.6	1.64 ± 0.11
4S-Fpr16	19.7 ± 3.2	4.78 ± 0.78
4R-Fpr16	3.2 ± 0.9	0.78 ± 0.22

Figure S1. Inhibition of cf-Api137 competing with unlabeled Api137 (black) and 4*S*-Fpr (red) and 4*R*-Fpr (blue) analogs for binding to the 70S ribosome. A, binding constant and K_d of cf-labeled Api137 to crude 70S ribosome. **B,** competitive binding of Fpr analogs with cf-labeled Api137. Error bars indicate the deviation from the mean in two independent experiments with two replicates. **C,** IC_{50} and K_i values calculated from the curves shown in panel B.

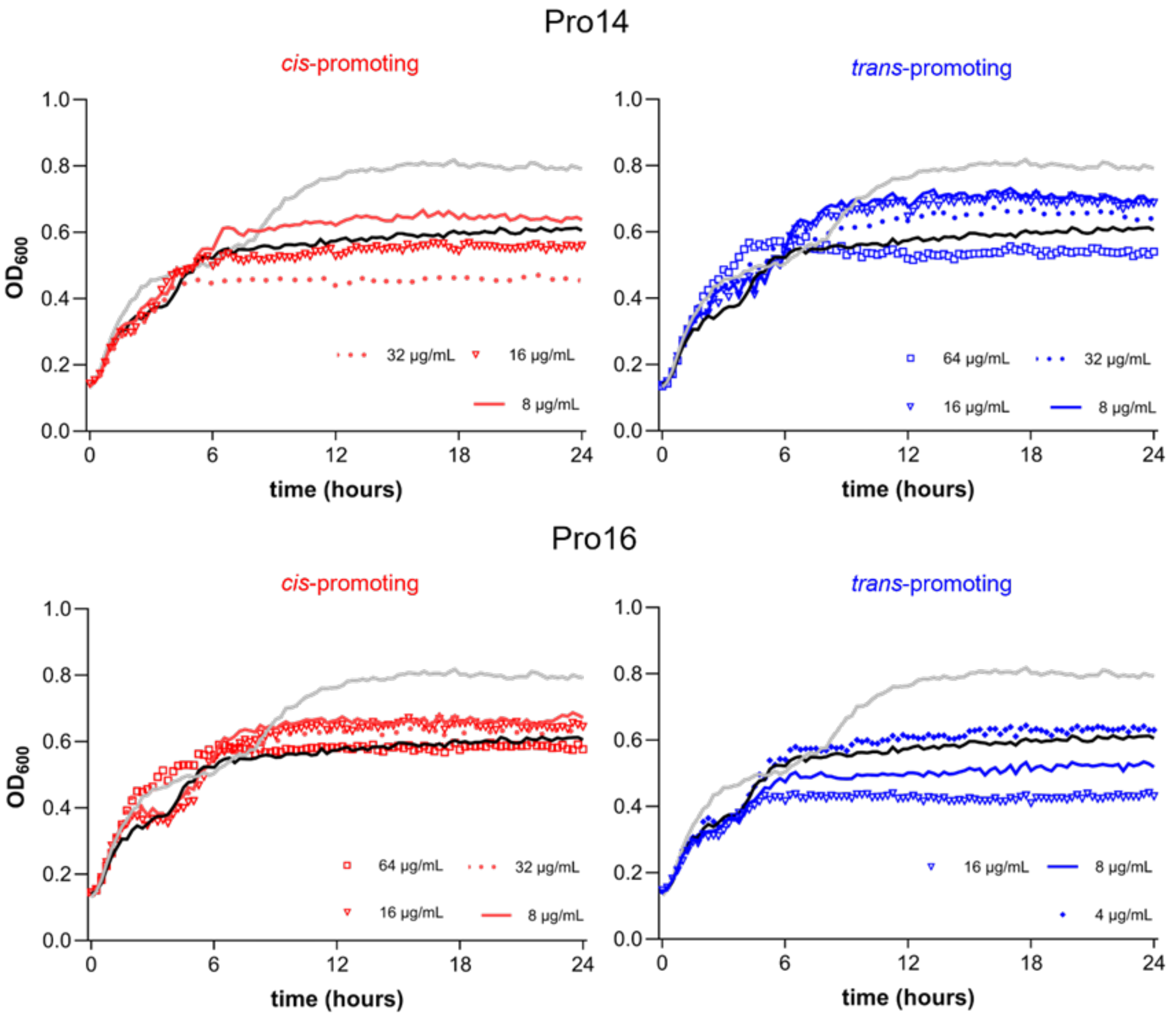

Figure S2. Growth curves of *E. coli* RN31 cultures incubated without PrAMP (light grey) and with Api137 (black, 8 µg/mL) and its analogs substituted with 4*S*-Fpr at positions 14 (top left) or 16 (bottom left) or 4*R*-Fpr at positions 14 (top right) or 16 (bottom right) at different concentrations. *E. coli* RN31 in the exponential growth phase (start OD_{600} 0.2) was added to a dilution series of PrAMPs in transparent 96-well plate and further cultivated for 24 h at 37 °C with 15 min measurement interval of OD_{600} in a PARADIGM^{TM} microplate reader (Beckman Coulter, Krefeld, Germany). The measurement was taken in two biological replicates.

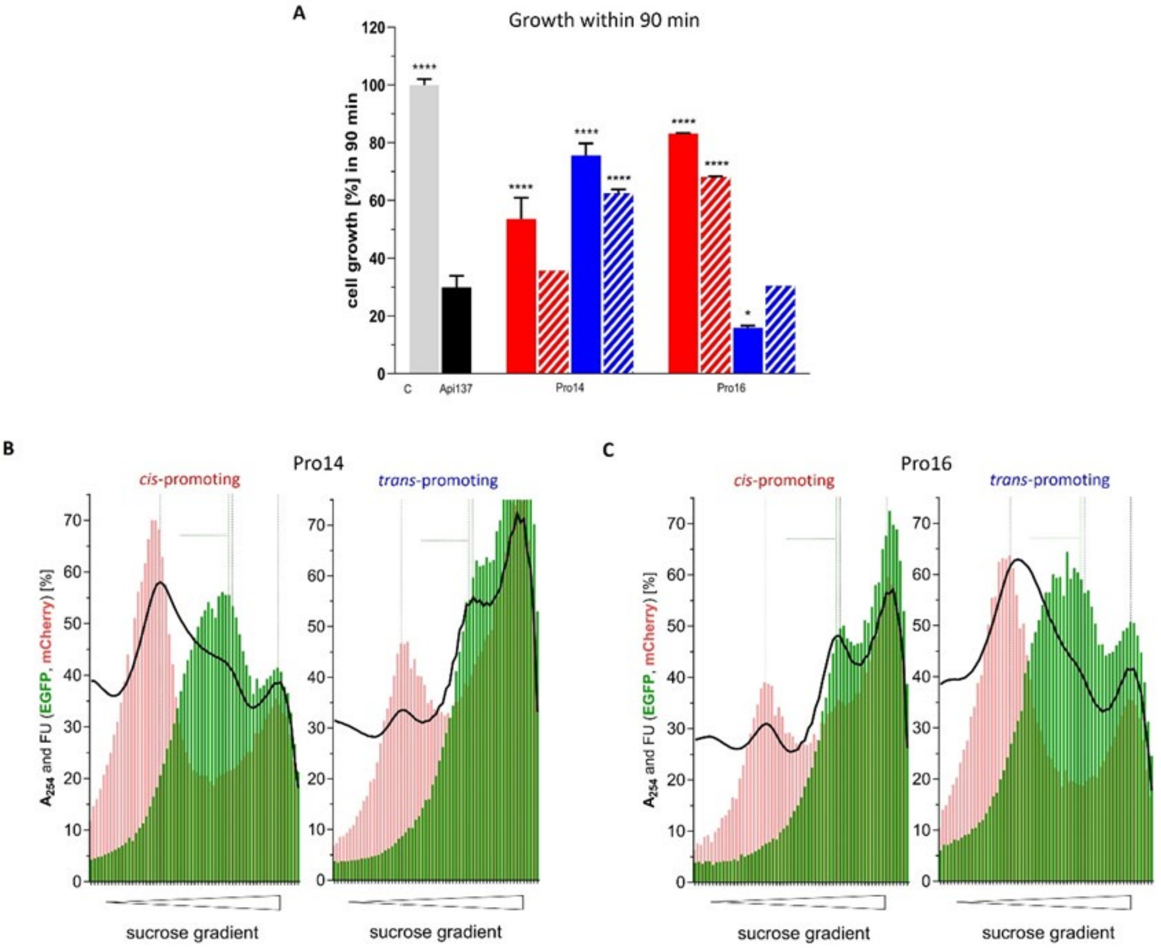

Figure S3. Growth rates of different PrAMPs (A) and ribosomal profiles of *E. coli* RN31 incubated with Api137 analogs substituted with 4*S*- (left) or 4*R*-Fpr (right) at positions 14 (B) or 16 (C). A, Cell growth inhibition of *E. coli* RN31 in 33% TSB after 90 min incubation without a PrAMP (gray, control = 100%) or in presence of Api137 (black) or an analog substituted by 4*S*-Fpr (red) or 4*R*-Fpr (blue) at the indicated positions. Full bars represent the results for PrAMP concentrations of 8 µg/mL and striped bars for the adjusted concentrations, namely 16 µg/mL for the 4*S*-Fpr14 analog (64% inhibition), 32 µg/mL for the 4*R*-Fpr14 (37% inhibition) and 4*S*-Fpr16 analogs (33% inhibition), 4 µg/mL for the 4*R*-Fpr16 analog (70% inhibition). Values were normalized to the OD_{600} of the control. Error bars indicate the deviation from the mean in two biological experiments. Significance levels versus Api137 are indicated as * (P value = 0.021) and **** (P value < 0.0001) by one-way ANOVA with Tukey's multiple comparison test (GraphPad Prism). **B, C,** *E. coli* RN31 expressing EGFP-bL19 (50S subunit, green bars) and mCherry-bS20 (30S subunit, red bars) were grown in 33% TSB medium and spiked with a PrAMP during the exponential growth phase. After 90 min, cells were lysed and separated by a 5-25% sucrose

gradient in TICO buffer. Shown are absorbance (λ = 254 nm) and fluorescence of EGFP (λ_{ex} = 485 nm, λ_{em} = 535) and mCherry (λ_{ex} = 585 nm, λ_{em} = 635 nm) for the fractions relative to the 70S maximum of the control (set to 100%). Dotted vertical lines indicate the maxima of the 70S, 50S, and 30S peaks in the control sample (Figure 3).

File S1: Materials and Chemicals

Reagents were obtained from the following companies unless stated otherwise: Biosolve BV (Valkenswaard, Netherlands): Acetonitrile (ULC/MS grade), formic acid (≥99%), *N,N*-dimethylformamide (DMF, > 99.8%), and piperidine (> 99.5%); Carl Roth GmbH & Co. (Karlsruhe, Germany): Dichloromethane (DCM, 99%), trifluoroacetic acid (TFA, ≥ 99.9%, peptide synthesis grade), lysozyme, sodium dodecyl sulfate (SDS), and glycerin (99.9%); Honeywell Fluka™ (Seelze, Germany): Ammonium bicarbonate (99.5%), 1,3-diisopropyl-carbodiimide (DIC, ≥ 98.0%), *N,N*-diisopropylethylamine (DIPEA, 98%), 1,2-ethandithiole (≥ 98%), $MgCl_2$ (99%), and thioanisole (≥ 98%); Sigma Aldrich (Steinheim, Germany): Ammonium chloride (NH_4Cl, 99.5%), 4-benzoyl-L-phenylalanine (BPA, 98%), 5(6)-carboxyfluorescein (Cf, for fluorescence), casein, *m*-cresol (98%), 1-hydroxy-benzotriazole hydrate (HOBt, ≥ 97.0%), hydrochloric acid (HCl), 2-mercaptoethanol (2-ME, 99%), *N*-methylmorpholine (NMM, 99.5%), potassium dihydrogen phosphate (KH_2PO_4, 99.5%), potassium hydroxide (KOH, 90%), sodium chloride (NaCl, 99.5%), sodium hydrogen phosphate (Na_2HPO_4, 99%), triisopropylsilane (TIS, >98.0%), and TFA (≥ 99%, HPLC grade); VWR International GmbH (Dresden, Germany): Acetonitrile (≥ 99.9%), diethyl ether (99.9%), and formic acid (FA, ~ 98%, LC-MS grade); AppliChem GmbH (Darmstadt, Germany): HEPES (99.5%) and tris(hydroxymethyl)aminomethane (TRIS); SERVA (Heidelberg, Germany): Acrylamide/bisacrylamide solution (37.5:1), ammonium persulfate (APS), Coomassie Brilliant Blue G250, tetramethylethylenediamine (TEMED, 99.9%), and Tween20; Iris Biotech GmbH (Marktredwitz, Germany): 2-(1*H*-benzotriazol-1-yl)-1,1,3,3-tetramethyluronium hexafluorophosphate (HBTU); Riedel-de Haën (Seelze, Germany): Bromophenol blue.

File S2: Mass spectra of the 4*S*-Fpr and 4*R*-Fpr substituted Api137 analogs

The identity of the Api137 analogs was confirmed by mass spectrometry. All singly protonated peptides have an exact mass of *m/z* 2309.3018. Api839 to Api844 were analysed by ESI-MS (esquire HCT Daltonics Ion Trap, Bruker) and Api845 to Api850 by MALDI-MS (MALDI TOF/TOF-MS 5800, AB Sciex).

Api839

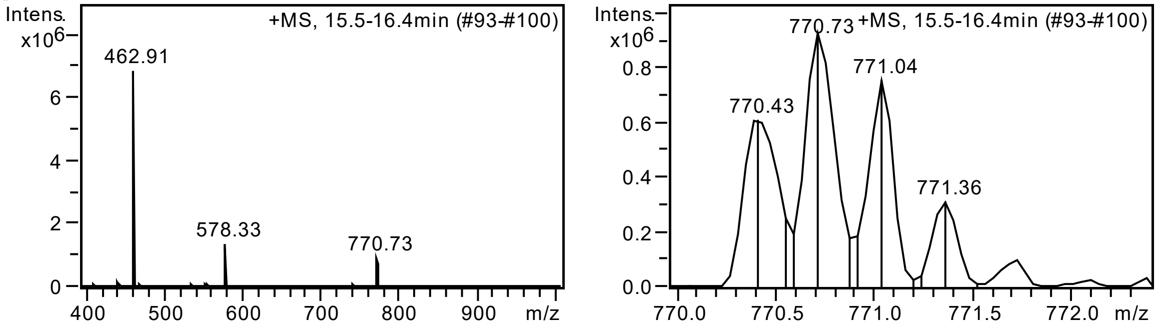

Api840

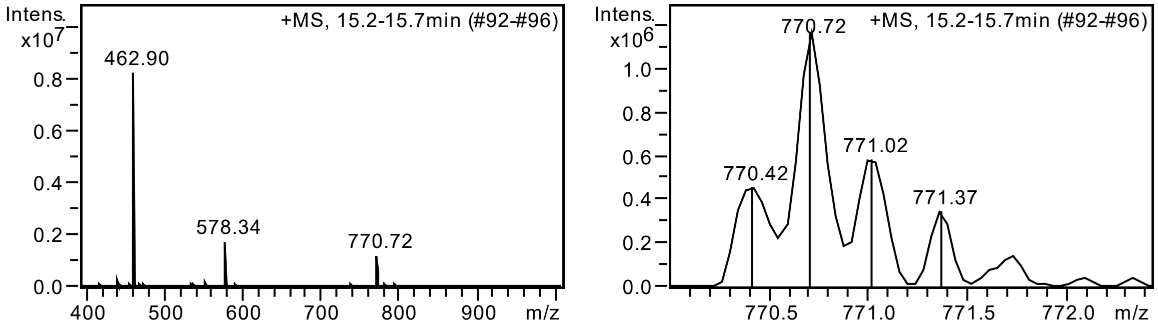

Api845

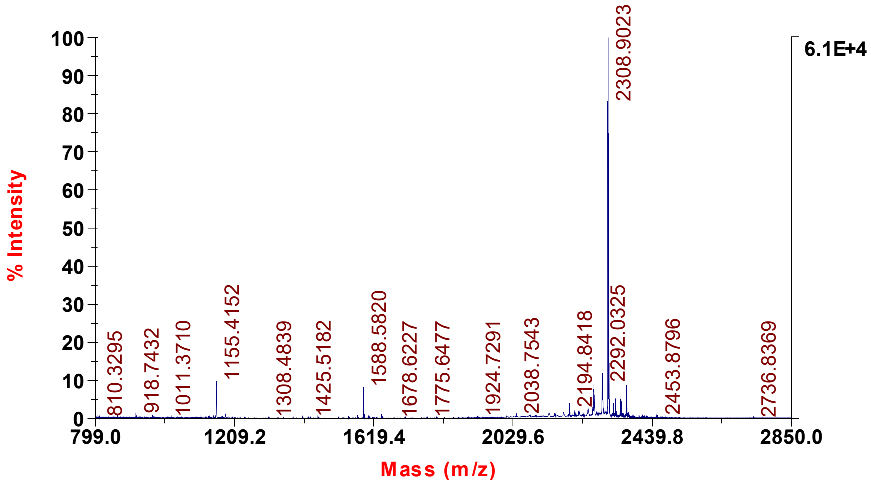

Api846

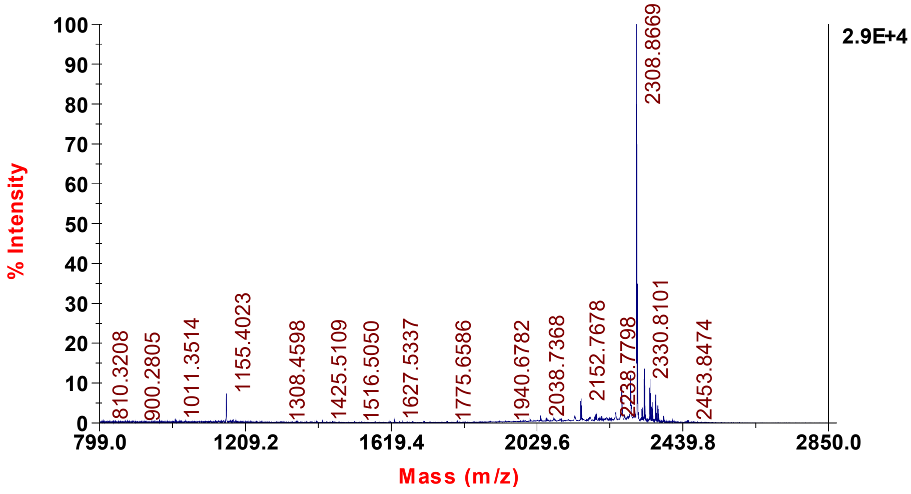

Api841

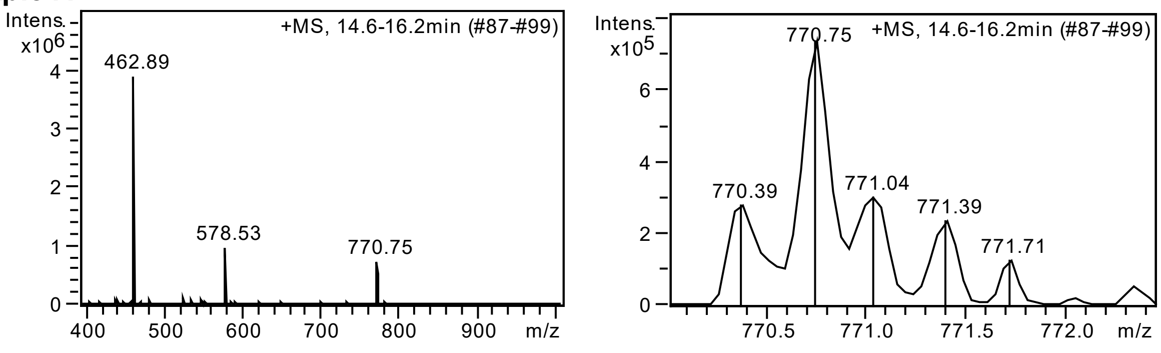

Api842

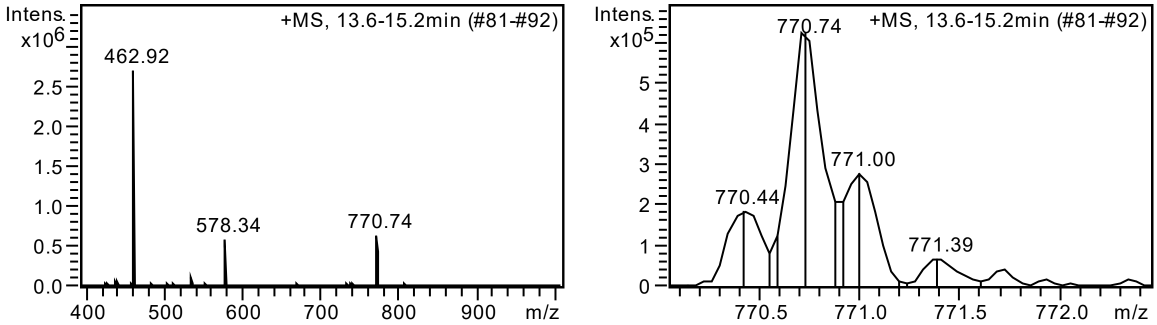

Api843

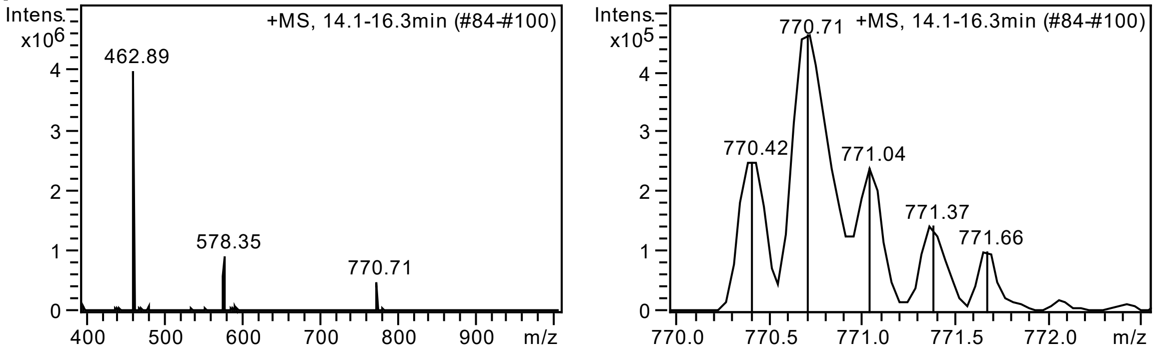

Api844

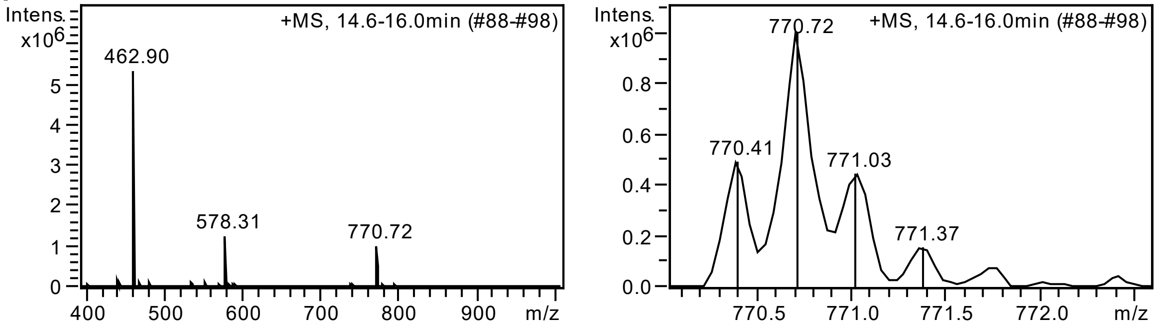

Api847

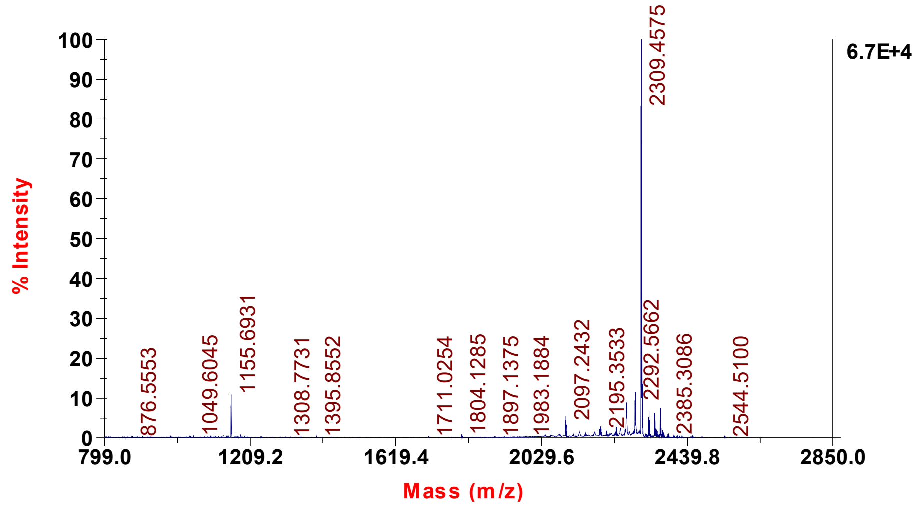

Api848

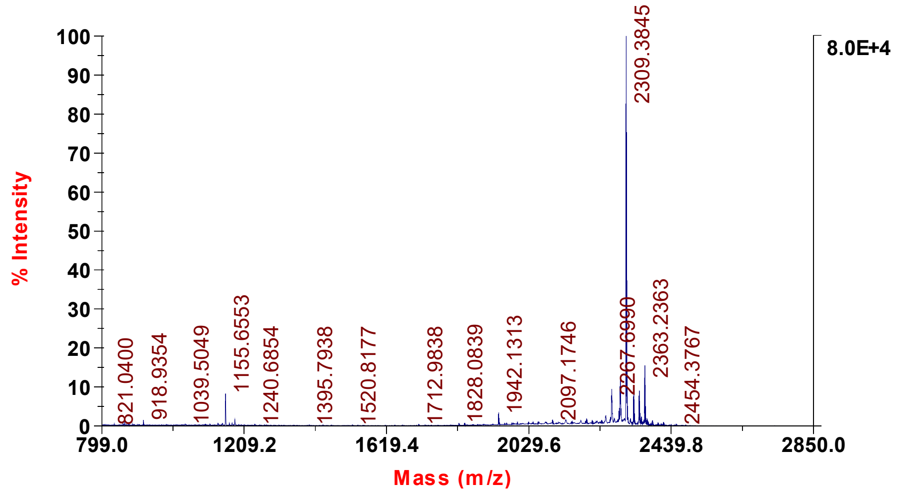

Api849

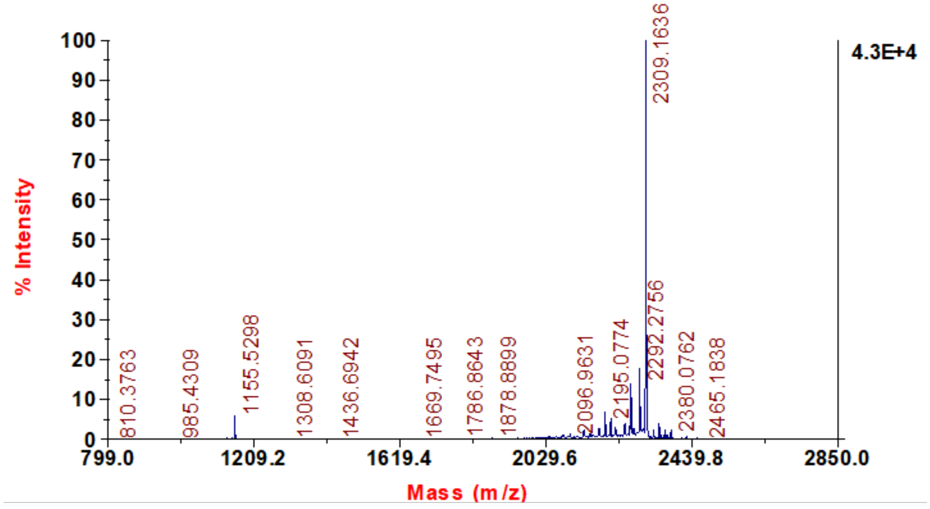

Api850

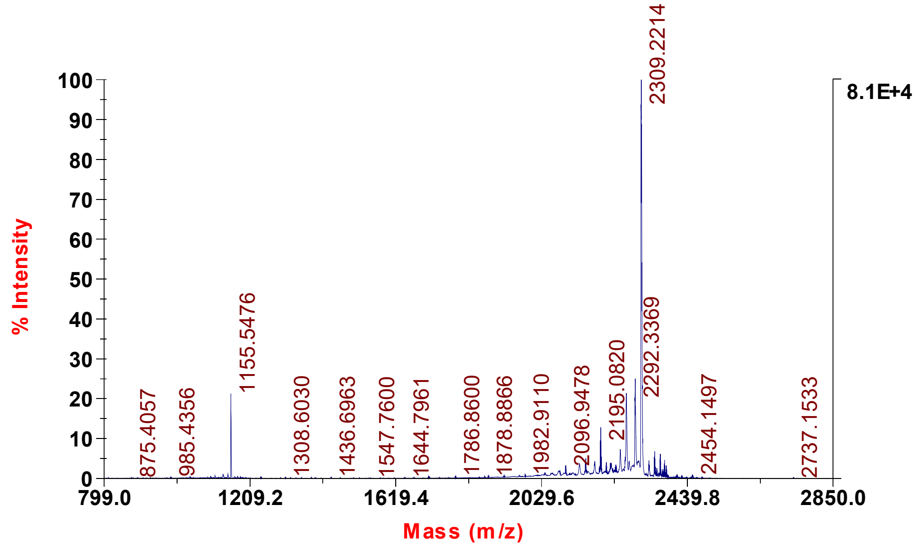

Supplement: Supplementary file 1 [file antibiotics-14-00566-s001.zip › antibiotics-3642063-supplementary.pdf]
